# Reply to Campbell et al. Comment on “Hogas et al. Salt, Not Always a Cardiovascular Enemy? A Mini-Review and Modern Perspective. *Medicina* 2022, *58*, 1175”

**DOI:** 10.3390/medicina59010083

**Published:** 2022-12-30

**Authors:** Mihai Hogas, Cristian Stătescu, Manuela Pădurariu, Alin Ciobica, Stefana Catalina Bilha, Anca Hăisan, Daniel Timofte, Simona Hogas

**Affiliations:** 1 Physiology Department, “Grigore T. Popa” University of Medicine and Pharmacy, Universitatii 16, 700115 Iasi, Romania; 2 Cardiology Department, “Grigore T. Popa” University of Medicine and Pharmacy, Universitatii 16, 700115 Iasi, Romania; 3 Psychiatry Department, “Grigore T. Popa” University of Medicine and Pharmacy, Universitatii 16, 700115 Iasi, Romania; 4 Department of Biology, Faculty of Biology, Alexandru Ioan Cuza University, B dul Carol I, no 11, 700506 Iasi, Romania; 5 Academy of Romanian Scientists, Splaiul Independentei nr. 54, sector 5, 050094 Bucuresti, Romania; 6 Center of Biomedical Research, Romanian Academy, Iasi, B dul Carol I, no 8, 700506 Iasi, Romania; 7 Endocrinology Department, “Grigore T. Popa” University of Medicine and Pharmacy, 700115 Iasi, Romania; 8 Surgery Department, “Grigore T. Popa” University of Medicine and Pharmacy, 700115 Iasi, Romania; 9 Academy of Medical Sciences Romania, 030167 Bucuresti, Romania; 10 Nephrology Department, “Grigore T. Popa” University of Medicine and Pharmacy, 700115 Iasi, Romania

We thank Campbell et al. for their comment [1] on our mini-review [2] of evidence regarding dietary salt intake and cardiovascular diseases (CVD).

We strongly agree with Campbell et al. [1] that reducing current salt intake is a desirable goal with undoubtable health benefits in the general population. The paper published by Hogas et al. [2] reviews current published data regarding the effect of dietary salt restriction upon various cardiovascular outcomes: the paper describes the valuable results of diet sodium reduction upon blood pressure (BP) values and the high cardiovascular risk associated with high sodium consumption. Far from intending to make any recommendations, Hogas et al. also review particular fragile situations in which dietary sodium reduction is employed, presenting data already published in the field [2]. Campbell et al. [1] remark that Hogas et al. [2] overlooked the consensus and global call to action published by Campbell et al. in May 2022 in the *Journal of Human Hypertension* [3]. However, the factsheet had not yet been published when data research performed by Hogas et al. ended [2]. Still, Hogas et al. fully agree that the current global sodium intake is too high, generating a high burden of disease from arterial hypertension and the increased cardiovascular risk that it generates, as already emphasized in the paper [2]. The meta-analysis [4] cited by Campbell et al. in their comment [1] reported a linear association between sodium intake and CVD, when the sodium intake is approximately between 2 g and 6 g per day. This was also reported by previous meta-analyses included in the mini-review of Hogas et al. [2] (Jayedi et al. [5], Strazzulo et al. [6], Aburto et al. [7]). As Campbell et al. recently state in the consensus published in 2022 [3], there is no established optimal level of sodium intake in order to reduce morbidity and mortality and thus, “optimal levels of sodium intake remain undefined”. In this context, controversial data are more prevalently reported when analyzing the effect of sodium restriction, rather than the effect of a high sodium diet ([8,9,10]). In addition, unexpected findings are often reported in specific patient categories [8], that are not representative of the general population.

Although controversial or “unexpected” data regarding non-classic findings of diet sodium restriction, such as the absence of a relevant curvilinear association between salt consumption and reduction in CVD-associated mortality in some papers [11], cannot be overlooked, limitations in the interpretation of these unusual findings were reviewed in the paper of Hogas et al. [2], and these include: the possibility of a concurrent diet rich in potassium, inconsistencies in study design among papers, patient life-style, etc. Despite the limitations of the Prospective Urban Rural Epidemiology (PURE) study already mentioned in the comment of Campbell et al. [1]—especially regarding urinary sodium excretion evaluation—the PURE study drew attention towards the very high global sodium consumption, with only a small proportion of patients adhering to the current recommendations [12].

In their recent paper, Cappuccio et al. [13] raise awareness towards misrepresentation of evidence supporting dietary sodium restriction. “Reverse epidemiology” has already been demonstrated for other general health risk factors (obesity improves survival in hemodialysis patients [14], smoking protects against endometrial cancer [15] and Parkinson’s disease [16]). This does not mean that inverse or “paradoxical” associations may be used as a rationale for promoting well-known health risk factors. However, reporting their paradoxical effects may help in elucidating some pathological mechanisms of the disease and lead to a more carefully tailored intervention.

The paper of Hogas et al. [2] totally supports the recommendations made by international scientific organizations and their guidelines and is not intended for guidance or advice. We agree that “it is of great public health importance to disseminate the best available evidence on dietary sodium” [1]. Narrative reviews, such as the paper of Hogas et al. [2], are intended for the presentation of existing knowledge on a topic based on all the published research available at the time of writing, including unusual or controversial findings, especially if published in top-rated journals (e.g., the European Heart Journal—an official journal of the European Society of Cardiology). Therefore, narrative reviews are not to be seen as equivalent to international guidelines and consensus, as they do not provide recommendations.

Campbell et al. further state in their comment [1] that the randomized controlled trial of Ezekowitz et al. [17] was not included in the paper of Hogas et al. [2] However, the paper was not yet available at the end of the literature research performed by Hogas et al. [2].

Finally, reducing dietary sodium—even slightly—is beneficial beyond doubt in the general population, while some reverse causality reports are still to be investigated and confirmed in specific populations. We agree with Campbell et al. that a population-wide approach would be beneficial, with salt-reduction strategies resulting in less people suffering from arterial hypertension and its consequences [13]. Reducing current salt consumption at a societal level would be a worthwhile contributor to strategies for reducing the worldwide burden of CVD [18].
